# Evaluation needs and temporal performance differences of gridded precipitation products in peripheral mountain regions

**DOI:** 10.1038/s41598-019-51666-z

**Published:** 2019-10-22

**Authors:** Harald Zandler, Isabell Haag, Cyrus Samimi

**Affiliations:** 1 Working Group of Climatology, Department of Geography, University of Bayreuth, Universitätsstr. 30, 95447 Bayreuth, Germany; 2 Bayreuth Center of Ecology and Environmental Research, University of Bayreuth, Dr. Hans-Frisch-Straße 1-3, 95448 Bayreuth, Germany

**Keywords:** Environmental sciences, Climate sciences, Climate change, Hydrology, Ecology, Climate-change ecology, Environmental social sciences, Climate-change impacts

## Abstract

Gridded datasets are of paramount importance to globally derive precipitation quantities for a multitude of scientific and practical applications. However, as most studies do not consider the impacts of temporal and spatial variations of included measurements in the utilized datasets, we conducted a quantitative assessment of the ability of several state of the art gridded precipitation products (CRU, GPCC Full Data Product, GPCC Monitoring Product, ERA-interim, ERA5, MERRA-2, MERRA-2 bias corrected, PERSIANN-CDR) to reproduce monthly precipitation values at climate stations in the Pamir mountains during two 15 year periods (1980–1994, 1998–2012) that are characterized by considerable differences in incorporated observation data. Results regarding the GPCC products illustrated a substantial and significant performance decrease with up to four times higher errors during periods with low observation inputs (1998–2012 with 2 stations on average per 124,000 km^2^) compared to periods with high quantities of regionally incorporated station data (1980–1994 with 14 stations on average per 124,000 km^2^). If independent stations were considered, the coefficient of efficiency indicated that only three of the gridded datasets (MERRA–2 bias corrected, GPCC, GPCC MP) performed better than the long term station mean for characterizing surface precipitation. Error patterns and magnitudes show that in complex terrain, evaluation of temporal and spatial variations of included observations is a prerequisite for using gridded precipitation products for scientific applications and to avoid overly optimistic performance assessments.

## Introduction

Atmospheric precipitation is a key environmental variable and the derivation of reliable rainfall amounts is of central importance for scientific research and a multitude of practical applications^1,2^. In many regions, gridded raster products are the only easily accessible and cost-free resources that provide information on surface precipitation compared to partly expensive or unavailable national gauge data collections. Generally, the different gridded datasets are categorized in gauge-based interpolations, satellite estimates, combinations of gauge and satellite data and reanalysis systems^2^. As an evaluation of respective products is necessary to assess their performance and related uncertainties, numerous studies exist that compare gridded precipitation values with gauge measurements of meteorological stations^3–14^. Although results vary, the Global Precipitation Climatology Centre (GPCC) full data product frequently outperforms other spatial rainfall estimates^6,8,9,15–19^. Therefore, GPCC or similar gauge-based raster datasets are frequently applied in many scientific disciplines. They are also used to assess climate change^20–23^ or to evaluate gridded rainfall products from other sources e.g.^24^. However, in spite of the fact that gauge based products originate from interpolated station values and statements that station data availability is considered to be a main influencing factor for the performance of these datasets^25–28^, the effect of regional and temporal station data variations was not considered in the majority of respective studies^8,9,16,17,19,20,29,30^. In peripheral regions, only some regional approaches over short time periods exist that indicate a substantial influence of incorporated observation data quantities on gauge dataset errors in Africa^7,12,31^. In Central Asian mountains, which are characterized by complex climate and immense topographically induced differences, only assumptions on the impact of data availability exist and in many post-soviet countries, gauge based validation data from more recent years, especially during and after the 1990s, is lacking e.g.^9^. This situation is exemplary for many other regions worldwide that experienced profound political or structural changes that influence meteorological measurement networks cf.^32^. Globally, extensive areas, including several main mountain ranges, are characterized by a lack of climate station data. Therefore, a comprehensive assessment of performance variations of respective precipitation datasets due to poor station networks and gauge data fluctuations is missing.

This study aims to address this research gap by evaluating several gridded precipitation datasets during two long-term periods. Thereby, the main objective is to assess time periods that are defined by a considerable difference of incorporated station data in the gauge-based products. We hypothesize that data availability has a major impact on the performance of associated precipitation estimates and respective datasets are not superior to other gridded products during periods of low station data availability. The Tajik Pamir region in Central Asia is an ideal example to evaluate this hypothesis: the available data for gauge based products dramatically dropped after the collapse of the Soviet Union and the area is characterized by complex terrain with two different precipitation regimes. Furthermore, it is of major importance for large scale hydrology, supra-regional water availability and it is a hotspot of climate change^33^. To cover different categories of precipitation datasets outlined in Sun *et al*.^2^ and based on their actuality, we include the *GPCC Full Data Monthly Product Version 2018*^34^, the *GPCC Monitoring Product Version 6*^35^, the climatic research unit (CRU) TS 4.03 dataset^36^, the *GPCP Version 2*.*3* product^37^, the *MERRA-2* and *MERRA-2-BC* products^38^, the *ERA-interim* product^39^, *ERA5*^40^, and the *PERSIANN-CDR* dataset^41^ for comparison of monthly precipitation amounts with ground based observation data from meteorological stations during the two 15 year periods 1980–1994 and 1998–2012. Additionally, we also evaluate the *TRMM 3B43* product^42^ during the latter period. Thereby, the *GPCC* and *CRU* products represent gauge-based datasets, the *GPCP Version 2*.*3* is a combination of satellite estimates and station observations, *TRMM 3B43* and *PERSIANN-CDR* are satellite-based products with additional station calibration or adjustment, and *ERA-interim*, *ERA5* and *MERRA-2* are reanalysis datasets. *MERRA-2* also offers a station based bias corrected version referred to as *MERRA-2 BC*. Various performance measures are calculated to shed light on temporal errors of the utilized datasets and their ability to provide atmospheric precipitation amounts in a complex peripheral mountain region. Thereby, we want to address potential issues of gridded precipitation products in peripheral, structurally weak mountain regions, the impacts of temporal variations of observational data and evaluation limitations.

## Study Area

**Figure 1:**
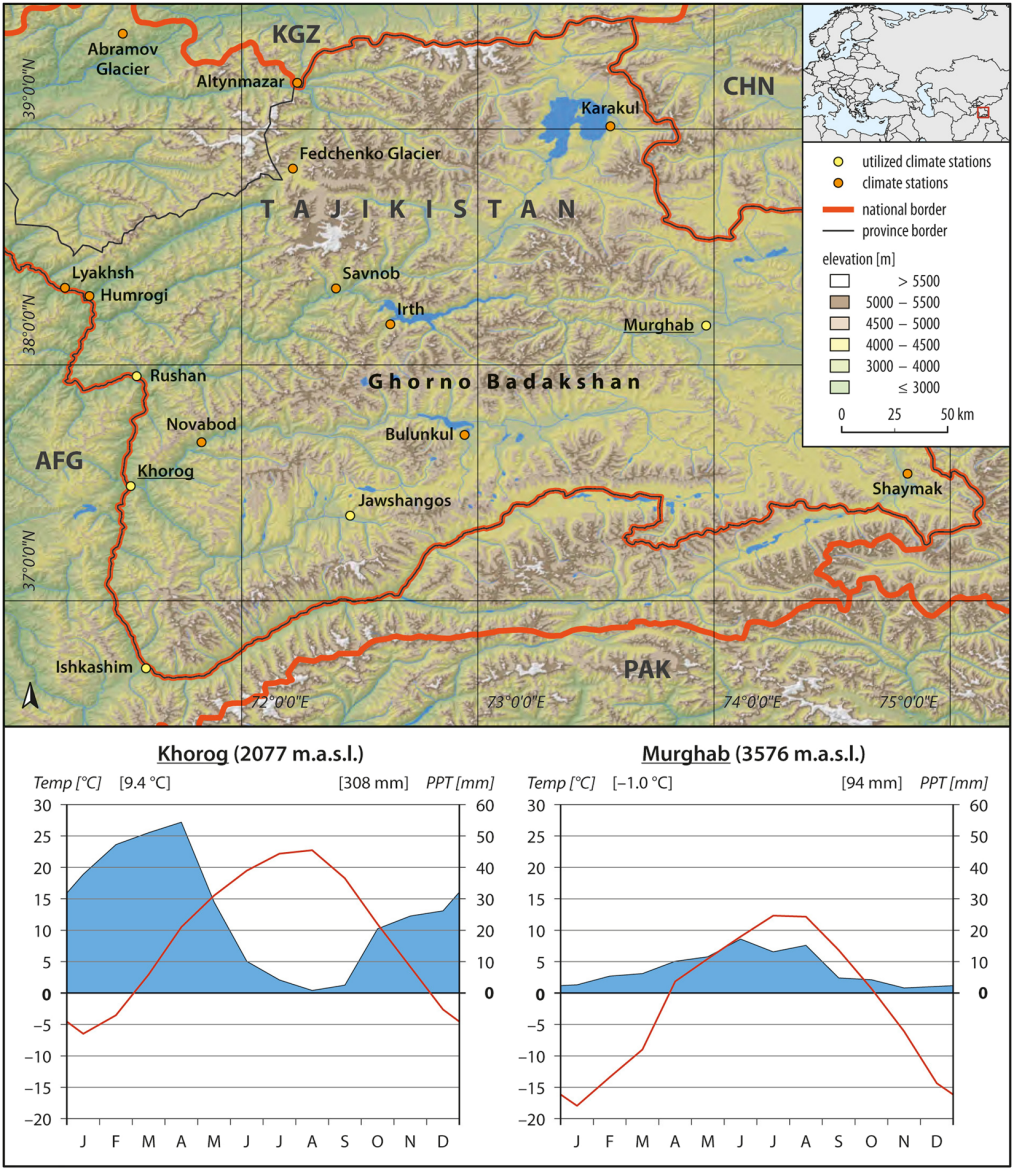
Overview of the study region with a total area of 124,000 km^2^, all regional climate stations with stations utilized for evaluation separately labelled, and climate diagrams for the period 1998–2012 at the locations Khorog and Murghab (Digital elevation model: SRTM^70^, vector data: DIVA-GIS^74^, meteorological data: SAHRT^47^).

The Pamir region of Tajikistan, largely synonymous to the political province Gorno-Badachschanskaja Avtonomnaja Oblast (Fig. 1), is a high mountain region with altitudes between 1,300 and 7,700 m.a.s.l. (mean 4,200 m.a.s.l.). The area is the intersection of several mountain ranges forming the source river (Panj) and upper watershed of a major Asian stream: the Amu Darya. This stream is of vital importance for large scale water supply as neighboring countries mainly depend on irrigated agriculture and benefit from solid water accumulation in high mountains during winter with a slow release in summer months^33^. Figures of an estimated water withdrawal of 63.1 km^3^ compared to a yearly average flow of 70 km^3^ demonstrate the significance of the Amu Darya for millions of people in the watershed, and it became internationally notorious by no longer reaching the Aral sea in the 1980s^43^. Because the water levels of the river and its delta are closely linked to the Pamir mountains^44^, precipitation variations in the region have an extensive and supra-regional hydrological impact. The area is topographically divided into a western part with deep valleys and strong elevation gradients and an eastern mountain plateau with a gentler topography at very high altitudes. In analogy to the topographic division, the climate also shows a dichotomous pattern. The western part is semi-arid with strong spring precipitation and dry summer months. The eastern part is characterized by a cold and arid climate with limited snow or rain amounts mostly falling during summer (Fig. 1).

**Figure 2:**
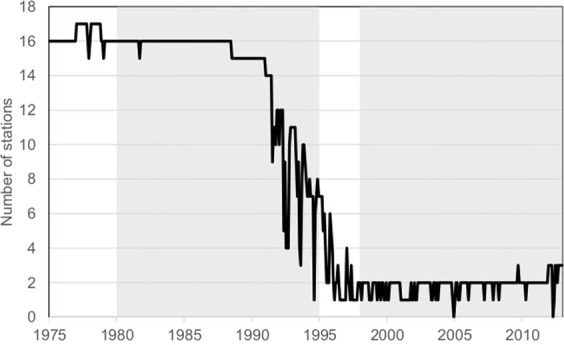
Time series of number of stations included in the GPCC Full Data Monthly Product in the research area from 1975–2012. Grey shaded areas show the two evaluation periods of this study.

The main reasons for this climatological divide is that the area forms the boundary between a zone dominated by the Westerlies in the west and a zone influence by the Indian summer monsoon in the southeast^13^. A similar division of the precipitation regime is exemplary for many regions in Central Asia, even though the major wind systems differ^19^. Although local climate observations started relatively early during the tsarist period in the 1890s and data quality was satisfactory until the 1990s, recent decades were characterized by a sharp decline in internationally available climate data due to the dissolution of the Soviet Union and the preceding civil war^33^. This is strikingly illustrated by station numbers regionally utilized in the *GPCC Full Data Monthly Product* which represents the largest precipitation data base of the world^26^ (Fig. 2). This situation renders the Pamir mountains as an ideal region to test the temporal and spatial performance of climatic precipitation datasets cf.^13^.

## Methods

Methods comprise the generation of a validation dataset from meteorological stations to test gridded precipitation products, the selection of relevant precipitation raster datasets and the calculation of error and performance measures. An additional evaluation of the impact of an automatic outlier correction algorithm addresses the potential of simple dataset improvement methods.

### Validation dataset

Observational datasets from different sources were combined to generate the longest possible temporal coverage for validation. A long term and frequently used dataset for Central Asia is provided from the National Snow and Ice Data Center (NSIDC) which ranges from 1879 to 2003 at some stations^45^. In the research area, station data from this dataset is only available until 1995. The same data is also available from the Northern Eurasian Earth Science Partnership Initiative (NEESPI) with temporal availability extended until 2007^46^. Consistency checks showed that monthly station values were identical to the NSIDC dataset. Therefore, we selected the NEESPI dataset for validation as it has better temporal coverage. For more recent periods, we directly acquired station data of eleven locations from the State Administration for Hydrometeorology of the Republic of Tajikistan (SAHRT)^47^ ranging from 1995 until 2012 at most stations. An examination of the agreement of the same stations during overlapping periods between NEESPI and SAHRT data resulted in identical values for most stations and months. A few months (n < 10, most in 1995) showed deviations between the datasets (e.g. different comma separators). In respective months, SAHRT data was selected as the correct value because respective dataset was directly obtained from the measuring authority. After compilation of the data, we excluded stations with less than 80% of available data in one of the observation periods according to World Meteorological Organization (WMO) criteria^48^. This left five meteorological stations for the validation process: Ishkashim, Jawshangos, Khorog, Murghab and Rushan (Fig. 1). Additional stations are located in the research area but these were excluded due to large data gaps.

### Gridded datasets

The selection of gridded precipitation products was based on their data availability from the 1980s until present or near present and on a global or near global scale. Other sources not providing data until present day or lacking updates at regular intervals (e.g. annually or biennially) were not considered. Furthermore, the selection was based on performance outlined in existing literature^3–14,49^. Finally, the evaluated datasets were intended to represent the following categories of gridded precipitation products to cover a broad methodological range: gauge-based interpolations, satellite data with gauge-based adjustments and reanalysis datasets. As a complete outline of the different algorithms used for creation of gridded datasets is out of scope of this study, a detailed description can be found in the cited literature and references therein. Only a short summary is presented in this section.

#### GPCC Full Data Monthly Product Version 2018

Referred to as *GPCC Full Data Product* in this study, this gauge-based dataset has a resolution of 0.25° and a temporal coverage from 1891 to 2016. It is considered as most accurate GPCC precipitation dataset^34^. The dataset includes a maximum of about 50,000 stations per month worldwide. Methodologically, precipitation anomalies of near-real time and non-near real time station data are interpolated using a long-term climatology. Schneider *et al*.^35^ provide a full description of all GPCC datasets.

#### GPCC Monitoring Product Version 6

Abbreviated *GPCC Monitoring Product* in this manuscript, this gauge-based dataset has a resolution of 1° and a temporal coverage from 1982 until two months before present^35^. It is limited to near real time station data from the Global Telecommunication System (GTS) of WMO and interpolates anomalies from the GPCC long-term climatology. This product was additionally included as it provides near real time data in contrast to the full data product.

#### CRU

The most recent version of the CRU dataset (TS 4.03) was released in 2019 and covers the time period from 1901 to 2018. It is based on meteorological stations, providing interpolated data values on a 0.5° longitude/latitude grid^36^. Observational records are derived from the WMO and the National Climatic Data Center (NCDC) databases. Temporarily, CRU includes more regional gauge observations before 1990.

#### GPCP Version 2.3

This product has a spatial resolution of 2.5° and a temporal coverage from 1979 to near present. It is based on merging infrared and microwave satellite estimates with various GPCC products^50,51^. Thereby, a two-step approach is applied. Satellite estimates are either multiplied by the large scale ratio of gauge analysis to the satellite results, or the differences of the large scale averages are added to the satellite estimates when gauge exceed satellite amounts^51^. Afterwards the gauge analysis and the gauge adjusted satellite product are merged using inverse-error-variance weighting. The product is designed to combine advantages of both satellite and gauge based approaches.

#### PERSIANN-CDR

This dataset has a resolution of 0.25° and covers the period from 1983 to near present. It uses GridSat-B1 IR satellite data and adjusts the high resolution data by using GPCP data at 2.5° resolution^52^. Thereby, satellite estimates are rescaled to GPCP resolution and a correction factor is calculated using the ratio of both products. After applying an optimization model to prevent unreasonably high corrections, the downscaled bias is removed from the satellite results. Temporarily, PERSIANN-CDR is characterized by low availability of included passive/active microwave observations before 1997^52^.

#### TRMM 3B43

The TRMM Multi-Satellite Precipitation Analysis Rainfall Estimate Product 3B43 Version 7 is characterized by a spatial resolution of 0.25° with a temporal coverage from 1998 until 2014. After the decommissioning of the TRMM satellite, the product was continued using climatological satellite calibrations resulting in a discontinuity in the data set^53^. The Integrated Multi-Satellite Retrievals for Global Precipitation Measurement (IMERG) replaces the product after retrospective processing and extends delivery of precipitation amounts until present. The TRMM product uses multiple microwave and infrared satellite precipitation estimates that are recalibrated with different GPCC datasets. Satellite data are adjusted using the large-scale means of the gauge analysis and combined applying an inverse estimated-random-error variance weighting^53^. In areas with high station number, the station values have a high effect on the resulting precipitation. However, in regions with poor gauge coverage such as the research area, the satellite input has much higher weight than the gauge adjustment^53^. Respective product was selected to represent more recent state of the art high-resolution satellite datasets.

#### MERRA-2

The Modern-Era Retrospective analysis for Research and Applications version 2 (MERRA-2) has a resolution of 0.5° × 0.625° and delivers data since 1980. It is an atmospheric reanalysis for the satellite era using the Goddard Earth Observing System Model, Version 5 with its Atmospheric Data Assimilation System^38^. Two datasets are used in this study: the original reanalysis version M*ERRA-2* and the bias corrected version, referred to as *MERRA-2 BC*, which uses NOAA Climate Prediction Center (CPC) Merged Analysis of Precipitation data at 2.5° and CPC Unified Gauge-Based Analysis of Global Daily Precipitation data at 0.5° resolution for correcting precipitation estimates^49^. The coarser resolution observation datasets are first downscaled to 0.5° and a correction factor is calculated as the ratio of observed to the modeled precipitation. It is then applied to the modeled values or in case of zero modeled precipitation, observed precipitation is added. Precipitation is finally calculated as weighted average of the corrected precipitation and precipitation of the atmospheric general circulation model^49^.

#### ERA-interim

*ERA-interim* covers the period from 1979 until present with a resolution of about 80 km (0.7° × 0.7°) on an irregular grid. It is a global atmospheric reanalysis products with a 12-hour analysis window and a four-dimensional variational analysis^39,54^. Available observations are thereby combined with a forecast model. The data assimilation process generates the global atmospheric situation and several physical parameters (such as precipitation) which are constrained by the available observations^54^. However, information on stations used for the assimilation is usually not available^55^. In contrast to gauge datasets, the European Reanalysis datasets are characterized by an increase in incorporated observational data in recent decades^54^.

#### ERA5

This reanalysis dataset is an enhancement of ERA-interim with a higher resolution of about 30 km (0.25° × 0.25°) on a regular grid starting in 1979. It is characterized by several methodological improvements compared to *ERA-interim*^56^. We included this product in addition to the older *ERA-interim* to evaluate potential performance increases.

### Observation periods

Data availability was the main criteria for defining our observation periods. The standard WMO climate period of three consecutive ten-year periods was not applicable in this study as data gaps exist. Furthermore, we used the shift in number of stations used in GPCC products during the 1990s for delimitation (Fig. 2). Gridded products cover different years and the most satellite based products still operational today started in the 1980s. Therefore, we selected the two 15-year periods 1980–1994 and 1998–2012. Exceptions were *PERSIANN-CDR* starting in 1983, the *GPCC Monitoring Product* starting in 1982 and *TRMM 3B43* with no data prior to 1998. Finally, we also included the years 1976–1990 for the *GPCC Full Data Product* to include error measures for a period characterized by very high station data availability in this dataset.

### Performance measures

Various upscaling or downscaling approaches exist to adapt or compare precipitation datasets of different resolutions and the selection of suitable methods depends on the specific application^57^. However, different techniques also lead to considerable variations of calculated performance measures^58^. To avoid associated uncertainties, we compared mentioned datasets in the original resolution and no rescaling algorithms were applied for the calculation of performance measures cf.^9^. This approach was also selected as respective products are frequently used without additional adjustments to derive local scale precipitation in applied scientific research. Finally, we consider the resolution of a dataset an important influencing parameter of product performance at the station scale and we want to originally include this information in our evaluation similar to existing research cf.^4,5,7,11^.

We selected measures for dataset evaluation based on their utilization in comparable studies and recommendations in the literature. The most widely applied measure is the coefficient of determination (*R²*) calculated as:

1 $${{\rm{R}}}^{2}={(\begin{array}{c}\underline{{\sum }_{n=1}^{n}({x}_{i}-\bar{x})({y}_{i}-\bar{y})}\\ \sqrt{{\sum }_{n=1}^{n}{({x}_{i}-\bar{x})}^{2}{\sum }_{n=1}^{n}{({y}_{i}-\bar{y})}^{2}}\end{array})}^{2}$$

where *n* is the number of values, *x*_*i*_ is the observed station value, *y*_*i*_ is the predicted value of the gridded dataset of month *i*, $\,\bar{x}$ is the mean of observed station values and $\bar{y}$ the mean of the gridded dataset values. However, the application of *R²* is limited as it depends on the data distribution, it is sensitive to outliers and proportional or additive errors are ignored^59^. Therefore, we also included Root mean squared error (*RMSE)*, Mean Absolute Error (*MAE)* and *BIAS* and their relative values in our evaluation using following formulas:

2 $${\rm{RMSE}}=\sqrt{\begin{array}{c}\underline{{\sum }_{{\rm{i}}={\rm{1}}}^{{\rm{n}}}{({x}_{i}-{y}_{i})}^{2}\,}\\ {\rm{n}}\end{array}}$$

3 $${\rm{RMSErel}}=\frac{{\rm{RMSE}}}{\bar{x}}\ast {\rm{100}}$$

4 $${\rm{MAE}}=\frac{1}{n}\,\mathop{\sum }\limits_{i=1}^{n}\,|{x}_{i}-{y}_{i}|$$

5 $${\rm{MAErel}}=\frac{{\rm{MAE}}}{\bar{x}}\ast {\rm{100}}$$

6 $${\rm{BIAS}}=\frac{1}{n}\,\mathop{\sum }\limits_{i=1}^{n}\,({y}_{i}-{x}_{i})$$

7 $${\rm{BIASrel}}=\frac{BIAS}{\bar{x}}\ast {\rm{100}}$$

In addition, we calculated the modified coefficient of efficiency (*Eff*). It is a dimensionless measure recommended for hydroclimatic evaluation ranging between minus infinity and one, whereby values below zero indicate that the observed mean station value is a better predictor than the predicted value of the gridded dataset^59^:

$${\rm{Eff}}=1-\begin{array}{c}\underline{{\sum }^{{\rm{n}}}\,|{x}_{i}-{y}_{i}|\,}\\ {\sum }_{{\rm{i}}=1}^{{\rm{n}}}\,|{x}_{i}-\bar{x}|\,\end{array}$$

Error measures were calculated with monthly values of all stations to derive overall performance of the gridded datasets. Additionally, error measures were also calculated for each station separately to illustrate spatial performance differences. This was implemented for both observation periods.

### Time series based outlier correction

Large outliers in precipitation products may lead to considerable deterioration of performance measures. To consider this effect in our analysis, we conducted an additional evaluation by applying a simple outlier correction algorithm to the grid based precipitation timer series data. We used the *tsclean* algorithm of the R based *forecast* package^60^ which replaces outliers greater than 1.5 interquartile range by a linearly interpolated value using a seasonally adjusted series.

### Homogeneity test

To assess if inhomogeneities are present in the datasets which may potentially affect the evaluation, we performed the homogeneity testing procedure proposed by Wijngaard *et al*.^61^ that was also applied by other evaluation studies^28^ during the whole 1980–2012 period. However, we used annual mean precipitation values instead of the annual wet day count (precipitation > 1 mm) because the latter was not available for most datasets. The methodology applies four tests and classifies the time series in the categories “useful”, “doubtful” and “suspect” according to respective results^62^. Additionally, three tests also outline the break periods.

### Significance tests

To test if there was a significant performance difference of the data sets between the two periods and between outlier corrected and original time series, we applied the non-parametric, paired, two-tailed Wilcoxon–Mann–Whitney test using the absolute differences between the station observations and the gridded values of each dataset.

## Results

### Climatological averages of gridded datasets

**Figure 3:**
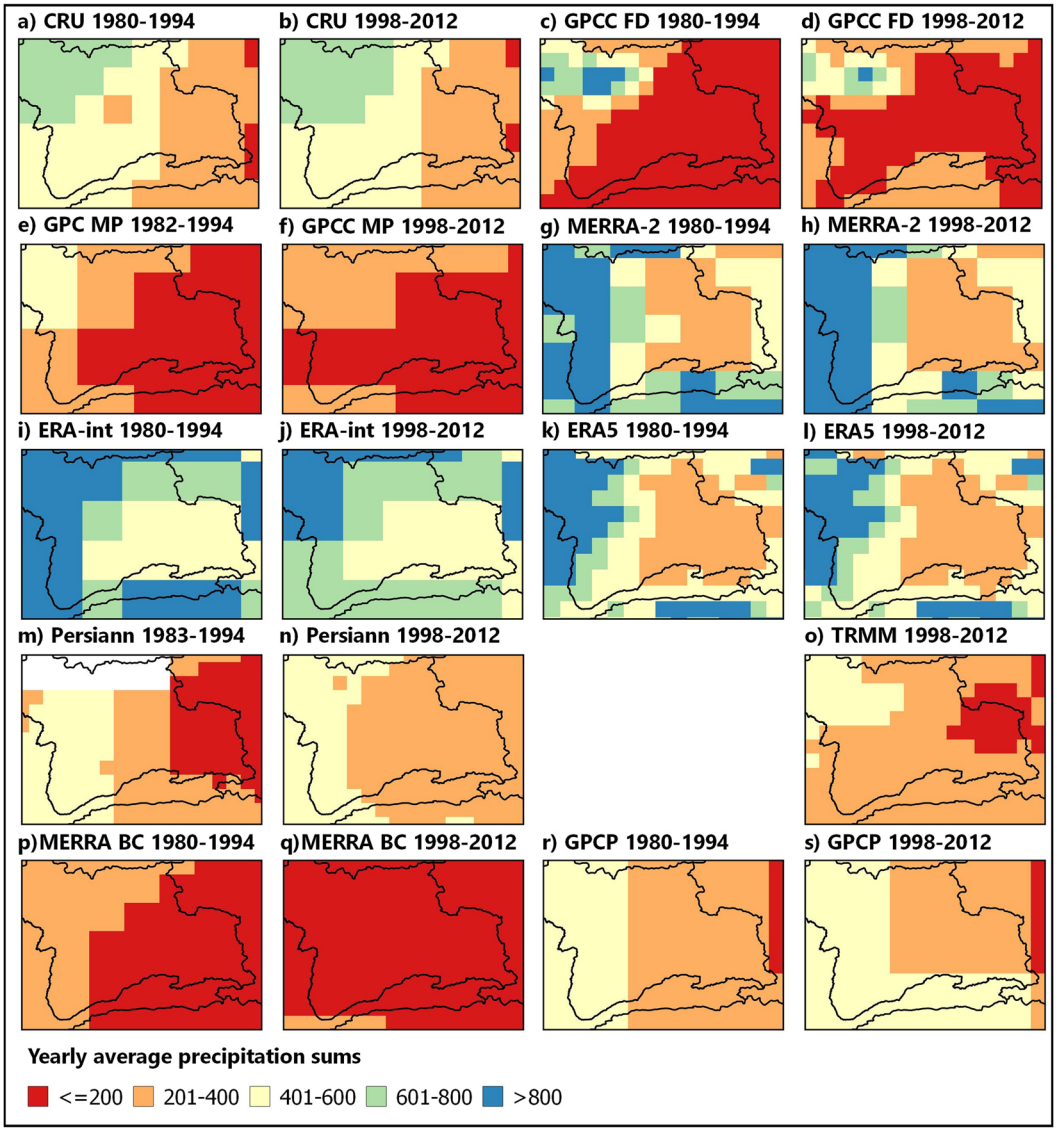
Climatological precipitation averages in mm for the periods 1980–1994 and 1998–2012 of station based (**a**–**f**), reanalysis (**g**–**l**), satellite based (**m**–**o**) and combined products (**p**–**s**). Please note that for visualization purposes, product acronyms used in the text were replaced by shorter versions if necessary. FD - Full Data Product, MP - Monitoring Product, ERA-int - ERA-interim, Persiann - Persiann-CDR and MERRA BC - MERRA-2 BC (Vector data: DIVA-GIS^74^, map created with: QGIS^75^).

All products displayed a general gradient of higher yearly precipitation values in the West and Northwest of the research area and lower values in the East (Fig. 3). Furthermore, several products exhibited higher sums in the southern high mountain ranges. Magnitudes between the different products showed differences with generally lower amounts of station based and combined products compared to much higher precipitation values in reanalysis datasets.

Satellite based products resulted in intermediate amounts. Temporarily, several products, such as MERRA-2 and ERA-interim indicated dryer conditions during the 1998–2012 period, whereas PERSIANN-CDR illustrated increased precipitation in the eastern part. Other products showed different patterns with an increase in some areas and decreases in other regions (e.g. GPCC Full Data Product).

### Homogeneity results

The homogeneity testing methodology indicated that most time series are homogenous and “useful”. The major exception was the MERRA-2 BC dataset with two time series at the stations classified as “suspect” and three classified as “doubtful”. Main breaks occurred during the years 1993–1996. PERSIANN-CDR and GPCP also showed “suspect” results for the Murghab location with breaks in 1997–2001. The GPCP Monitoring Product resulted in a “doubtful” time series for the Ishkashim location with a break in the first year.

### Performance of datasets

Performance measures showed strong differences between the various products and observation periods. From 1998–2012, *Eff* indicates that only four of the nine datasets performed better than the mean station value in estimating monthly precipitation amounts (Table 1). Similarly, only these dataset resulted in a lower *MAE* than the mean observed precipitation of about 17 mm per month averaged over all stations in this period. The best performing dataset during 1998–2012 in terms of *Eff*, *MAE* and *RMSE* was the bias corrected reanalysis product *MERRA-2 BC*. However, the error measures of the *GPCC* datasets were very similar. Satellite based *TRMM* resulted in slightly higher errors. All other datasets indicated very high errors in this period. Results for *R²* were different with highly significant values of all datasets (p < 0.001) but highest correlation values of the reanalysis products.

**Table 1:** Performance measures of gridded datasets compared to station observations during the period 1998–2012.

Dataset	R²	p	RMSE	RMSErel	MAE	MAErel	BIAS	BIASrel	Eff
MERRA-2 BC	0.35	<0.001	**19**.**11**	**109**.**28**	**11**.**61**	**66**.**40**	−4.11	−23.52	**0**.**29**
GPCC Full Data Product	0.27	<0.001	23.35	133.55	13.17	75.29	−**2**.**30**	−**13**.**13**	0.20
GPCC Monitoring Product	0.26	<0.001	22.99	131.49	13.66	78.13	−3.66	−20.92	0.17
TRMM	0.35	<0.001	23.82	136.22	15.32	87.62	7.68	43.90	0.07
PERSIANN-CDR	0.38	<0.001	31.45	179.87	21.13	120.82	17.55	100.37	−0.29
GPCP	0.30	<0.001	36.23	207.20	26.78	153.13	23.95	136.95	−0.63
CRU	0.34	<0.001	39.81	227.68	30.16	172.45	27.65	158.11	−0.84
ERA-interim	0.53	<0.001	47.83	273.55	38.43	219.75	38.10	217.87	−1.34
ERA5	0.54	<0.001	52.31	299.14	41.00	234.45	40.40	231.02	−1.50
MERRA-2	**0**.**57**	<0.001	67.77	387.58	49.12	280.88	48.60	277.92	−2.00

Products are ascendingly sorted based on *MAE* values and best dataset is indicated in bold text respectively. Resolutions of products are as follows: GPCC Full Data Product: 0.25°. GPCC Monitoring product: 1°, CRU: 0.5°, GPCP: 2.5°, PERSIANN-CDR: 0.25°, TRMM: 0.25°, MERRA-2 and MERRA-2 BC: 0.5° × 0.625°, ERA-interim: 0.7° irregular and ERA5: 0.25°.

**Figure 4:**
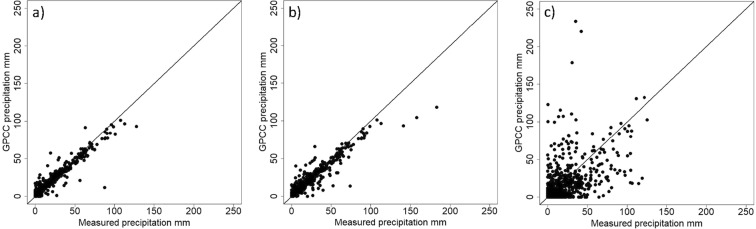
Scatterplots with superimposed 1:1 line between monthly precipitation values measured at the meteorological stations (x-axis) and amounts derived from *GPCC Full Data Product* (y-axis) during the periods (**a**) 1976–1990, (**b**) 1980–1994 and, (**c**) 1998–2012.

During the 1980–1994 period, the *GPCC Full Data Product* clearly outperformed all other datasets in estimating station precipitation amounts with lowest errors and better performance measures compared to all other datasets (Table 2). *MERRA-2 BC* and the *GPCC Monitoring Product* also showed relatively good performance with a positive *Eff* and a better *MAE* compared to the monthly mean observed precipitation of about 15 mm averaged over all stations. Similarly to the period 1998–2012, the other raster products showed low performance measures and high error values. R² showed comparable values of the reanalysis products to the 1998–2012 period. Regarding differences in the performance during the two periods, the *GPCC* datasets and the *MERRA-2 BC* product showed a strong decrease in performance in the more recent period 1998–2012 which was very highly significant in terms of absolute differences (Table 3). The *GPCC Full Data Product* resulted in a fourfold increase of errors with a growth of about 10 mm in *MAE* and a more than threefold increase of *RMSE* with a growth of 17 mm. *MAErel* resulted in a rise of 54 percentage points with this product. The reanalysis datasets *ERA-interim* and *ERA5*, and the satellite derived *PERSIANN-CDR* product showed an increase in performance measures in 1998–2012 with a very highly significant decrease (p < 0.001) of absolute differences between station data and gridded estimates. Largest improvements were visible for *ERA-interim* and *PERSIANN-CDR*. The period from 1976–1990 showed lowest error estimates for the *GPCC Full Data Product*, the only evaluated dataset during this time, with an *Eff* of 0.82, *MAE* of 2.49, a *RMSE* of 5.31 and a *R²* of 0.93. This corresponds to a fivefold lower *MAE* compared to 1998–2012. Scatterplots of *GPCC Full Data Product* precipitation and sums measured at stations showed considerable differences between the two earlier periods and the latest period (Fig. 4).

**Table 2:** Performance measures of gridded datasets compared to station observations during the period 1980–1994.

Dataset	R²	p	RMSE	RMSErel	MAE	MAErel	BIAS	BIASrel	Eff
GPCC Full Data Product	**0**.**90**	<0.001	**6**.**67**	**44**.**91**	**3**.**21**	**21**.**64**	−**0**.**19**	−**1**.**30**	**0**.**77**
MERRA-2 BC	0.44	<0.001	17.42	117.35	9.23	62.19	3.22	21.69	0.35
GPCC Monitoring Product	0.27	<0.001	23.23	158.62	9.74	66.51	3.63	24.77	0.31
PERSIANN-CDR	0.41	<0.001	38.16	248.39	25.54	166.24	22.57	146.94	−0.75
GPCP	0.37	<0.001	36.68	247.08	27.13	182.73	25.57	172.23	−0.91
CRU	0.50	<0.001	38.20	257.30	29.63	199.55	28.63	192.85	−1.08
ERA5	0.54	<0.001	55.44	373.45	43.95	296.03	43.70	294.34	−2.09
ERA-interim	0.54	<0.001	56.14	378.17	46.31	311.92	46.11	310.57	−2.26
MERRA-2	0.54	<0.001	66.21	445.97	48.08	323.86	47.66	321.05	−2.38

Products are ascendingly sorted based on *MAE* values and best dataset is indicated in bold text respectively. Resolutions of products are as follows: GPCC Full Data Product: 0.25°. GPCC Monitoring product: 1°, CRU: 0.5°, GPCP: 2.5°, PERSIANN-CDR: 0.25°, TRMM: 0.25°, MERRA-2 and MERRA-2 BC: 0.5° × 0.625°, ERA-interim: 0.7° irregular and ERA5: 0.25°.

**Table 3:** Differences of selected performance measures between the periods 1998–2012 and 1980–1994.

Dataset	R²	RMSE	MAE	BIAS	Eff
GPCC Full Data Product	−**0**.**63**	**16**.**68**	**9**.95***	−2.10	−**0**.**58**
GPCC Monitoring Product	−0.01	−0.24	3.92***	−7.29	−0.14
MERRA-2 BC	−0.09	1.69	2.38***	−7.33	−0.06
CRU	−0.17	1.61	0.53	−0.98	0.24
MERRA-2	**0**.**03**	1.56	1.03	**0**.**93**	0.39
GPCP	−0.07	−0.45	−0.35	−1.62	0.28
ERA5	0.00	−3.14	−2.95***	−3.30	0.59
PERSIANN-CDR	**0**.**03**	−6.7	−4.41**	−5.02	0.47
ERA-interim	−0.01	−**8**.**31**	−**7**.88***	−**8**.**01**	**0**.**91**

Relative performance measures were excluded as they are also influenced by the difference of the period averages. Largest decreases and increases in performance are indicated in bold text. Asterisks after the MAE value indicate the significance level of absolute differences (none - not significant, **-highly significant (p < 0.01), and ***- very highly significant (p < 0.001)).

**Figure 5:**
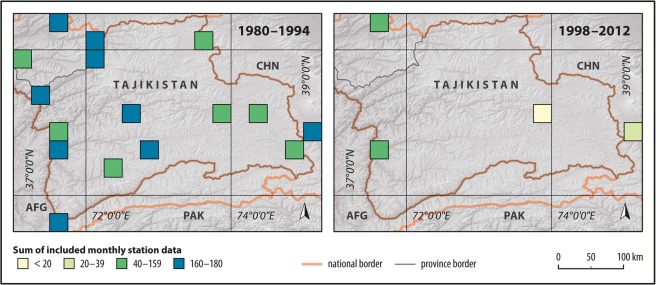
Summed number of included gauges per grid cell in the *GPCC Full Data Product* during the periods 1980–1994 (left) and 1998–2012 (right). E.g. one station continuously included every month in one pixel during a whole period would result in a total of 180 (Digital elevation model: SRTM^70^, vector data: DIVA-GIS^74^).

The geographical distribution of incorporated station data in GPCC Full Data Product illustrated strong differences between the two periods and a shift from roughly uniformly distributed gauge data availability during 1980–1994 to a situation with only two frequently available stations during 1998–2012 (Fig. 5).

All stations used for evaluation were at least partly included in the GPCC dataset during the 1980–1994 period as well. From 1998 to 2012, only Khorog station data was regularly included in the GPCC product. All other evaluation stations were not included in the GPCC dataset with the exception of Murghab station, which was included for one month only. The CRU dataset only includes data from Khorog station after 1990 whereby the available data strongly decreased from the 1980–1994 period to the 1998–2012 period^36^. Before 1990, partly erratic data was also included for the evaluated stations Ishkashim, Jawshangos, Murghab and Rushan. To additionally assess the bias of congruent station data, we also conducted an analysis excluding Khorog station from the evaluation during the 1998–2012 period. Evaluation differences between the datasets with the four remaining independent stations and the original evaluation dataset resulted in a performance decline of most products (Table 4). ERA-interim showed highest increases with 39 percentage points in MAErel. MERRA-2 was the only dataset that showed slightly increased performance after the removal of Khorog station. Only three datasets (MERRA-2 BC, GPCC Full Data Product, GPCC Monitoring Product) indicated better performance than the mean station value as given by *Eff* with the reduced evaluation set. The relative order based on MAE remained similar without the Khorog evaluation station.

**Table 4:** Performance measures of gridded datasets compared to station observations excluding Khorog station during the period 1998–2012.

Dataset	R²	p	RMSE	RMSErel	MAE	MAErel	BIAS	BIASrel	Eff
MERRA-2 BC	0.28	<0.001	**17**.**88**	**118**.**67**	**11**.**01**	**73**.**06**	−2.06	−13.65	**0**.**21**
GPCC Full Data Product	0.20	<0.001	23.46	155.70	13.14	87.23	−**0**.**68**	−**4**.**52**	0.06
GPCC Monitoring Product	0.21	<0.001	22.50	149.33	13.26	87.99	−1.91	−12.68	0.05
TRMM	0.30	<0.001	24.08	159.83	15.41	102.27	9.24	61.30	−0.11
PERSIANN-CDR	0.33	<0.001	32.38	214.89	21.69	143.94	18.92	125.55	−0.56
GPCP	0.08	<0.001	38.67	256.62	28.40	188.49	23.71	157.34	−1.04
CRU	0.30	<0.001	40.50	268.82	30.62	203.23	28.46	188.87	−1.20
ERA5	0.48	<0.001	50.64	336.06	37.99	252.11	37.23	247.11	−1.73
ERA-interim	**0**.**50**	<0.001	48.51	321.93	38.94	258.41	38.69	256.80	−1.79
MERRA-2	0.47	<0.001	57.30	380.30	41.99	278.68	41.37	274.54	−2.01

Products are ascendingly sorted based on *MAE* values and best dataset is indicated in bold text respectively.

**Figure 6:**
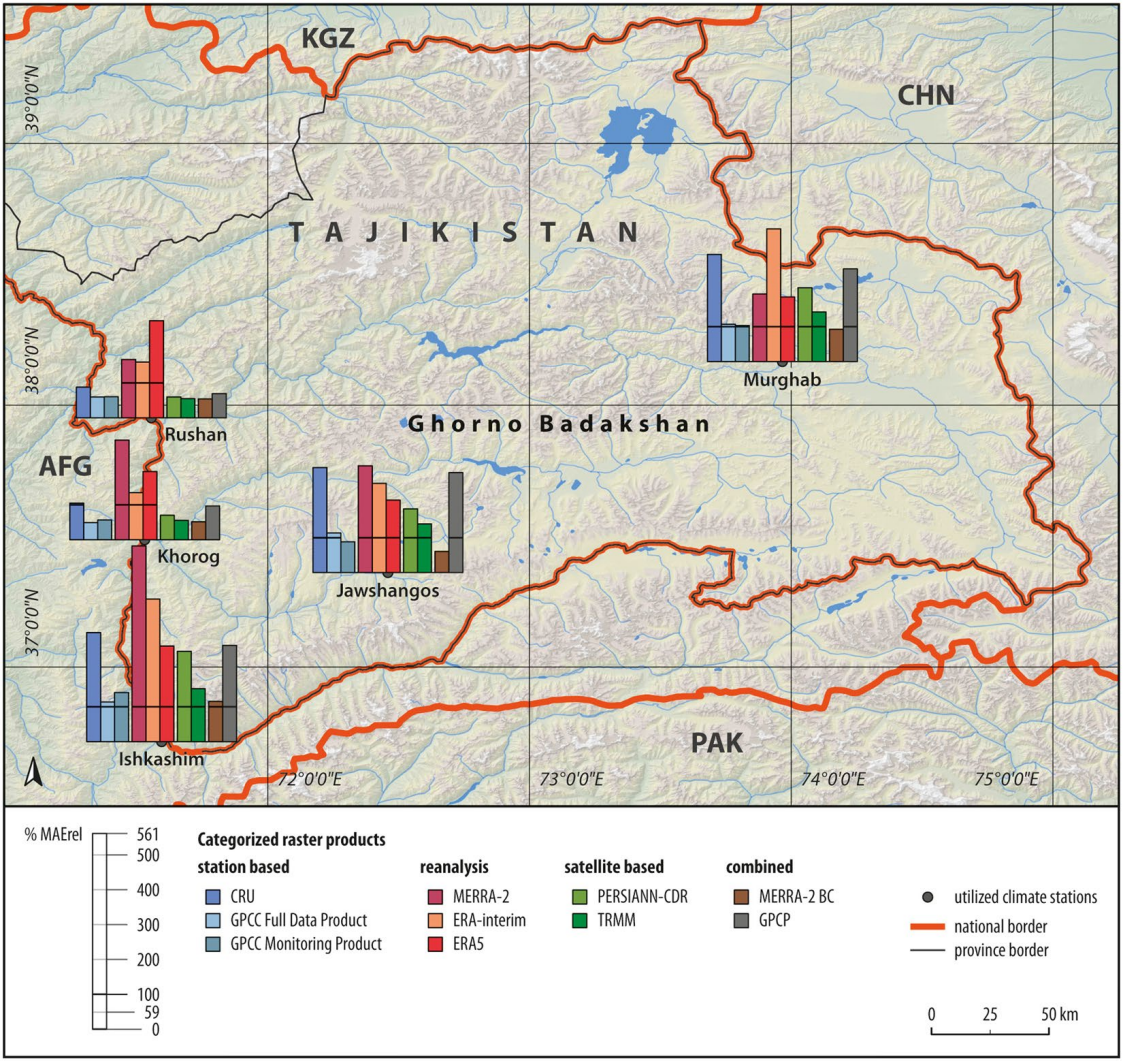
Spatial distribution of *MAErel* at the different stations during the 1998–2012 period. Horizontal black line in diagrams indicates *MAErel* of 100**%** (Digital elevation model: SRTM^70^, vector data: DIVA-GIS^74^).

The spatial assessment of the relative *MAE* of station based, satellite based and combined products showed a pattern of lower errors in Khorog and Rushan compared to much higher values at the other locations during the 1998–2012 period (Fig. 6). With the exception of reanalysis datasets, these two locations also resulted in *MAErel* values below 100% compared to most other locations. Highest errors were visible in Ishkashim and to a lesser extent, in Murghab in the East.

### Outlier correction impacts

The outlier correction algorithm showed an improvement of some datasets in the 1998–2012 period with a decrease of relative *RMSE* of up to 31 percentage points (*MAErel*: 12 percentage points) with the *GPCC Full Data Product* (Table 5). However, only two other products (*GPCC Monitoring Product*, *TRMM*) resulted in apparent improvements due to outlier correction in this period. During the 1980–1994 period, largest performance increases were visible for *GPCC Monitoring Product* with relative *RMSE* values reduced by 64 percentage points. *MERRA-2 BC* and *GPCP* also showed some improvement after the outlier correction but for the *GPCC Full Data Product*, performance measures worsened due to the algorithm.

**Table 5:** Differences of selected performance measures between the outlier corrected datasets and the original gridded datasets during the periods 1998–2012 and 1980–1994.

Dataset	1998–2012				1980–1994			
	RMSE	RMSErel	MAE	MAErel	RMSE	RMSErel	MAE	MAErel
CRU	0.00	0.00	0.00	0.00	0.00	0.00	0.00	0.00
ERA-interim	0.00	0.00	0.00	0.00	0.00	0.00	0.00	0.00
ERA5	−0.29	−1.64	−0.38*	−2.20	−0.06	−0.38	−0.10	−0.66
GPCC Full Data Product	−**5**.**45**	−**31**.**19**	−**2**.04***	−**11**.**69**	3.42	23.06	0.49**	3.32
GPCC Monitoring Product	−2.83	−16.17	−1.15**	−6.55	−**9**.**36**	−**63**.**94**	−**1**.43*	−**9**.**73**
GPCP	−0.08	−0.47	−0.06	−0.36	−1.54	−10.35	−0.48	−3.26
MERRA-2	0.00	0.00	0.00	0.00	−0.04	−0.25	−0.06	−0.43
MERRA-2 BC	−0.53	−3.04	−0.21	−1.20	−3.78	−25.44	−0.77*	−5.18
PERSIANN-CDR	0.00	0.00	0.00	0.00	0.1	−5.71	0.05	3.7
TRMM	−3.34	−19.11	−1.42***	−8.12				

Largest differences in each period are indicated in bold text. Asterisks after the *MAE* value indicate the significance level of changes in absolute differences (none - not significant, *- significant (p < 0.05), **-highly significant (p < 0.01) and ***- very highly significant (p < 0.001)).

## Discussion

This is the first study that evaluates and quantifies temporal and spatial performance differences of gridded precipitation products over two long term periods in peripheral high mountains. Results clearly showed that gauge data availability is one of the main issues that has to be taken into account before using respective products for subsequent research or planning applications. In periods of poor station data inputs ($\varnothing$ 2 stations/124,000 km^2^), monthly errors (*MAE*) of the *GPCC Full Data Product* increased by a factor of four compared to time periods with good station data coverage ($\varnothing$ 14 stations/124,000 km^2^). In relative terms, this corresponds to an increase in *MAErel* of 54 percentage points or more than half of the long term monthly gauge average. Although this result was not unexpected because station density was important for the reliability of gridded precipitation products in previous research^25,63^, we quantitatively document the potential magnitude of error increases in complex terrain in contrast to studies that utilize station based gridded datasets without considering the number of incorporated climate stations in their research e.g.^19,21–23,29^. Moreover, existing evaluation studies indicating a temporal decline in correlation between station data and gridded products did not include an associated analysis of weather stations incorporated in the used datasets^9^. However, our results provide evidence that missing station data in gridded rainfall products may cause errors that are too large for a meaningful analysis of long term environmental change as they only provide slightly better values of monthly precipitation at the stations than long term station averages. The very good performance of the *GPCC Full Data Produc*t until 1994, a period during which station data was included in the dataset at all our validation locations, also showed that ignoring the position, the number and the temporal variation of incorporated gauge measurements may result in overly optimistic evaluation results as the same or proximate stations may be applied for evaluation of gridded products that are also used for creating the datasets. Our findings demonstrate that studies which utilize respective data and lack associated assessments have to be treated with caution. In the context of Central Asia, existing research analyzed larger areas whereby validation data was largely not available after the late 1990s for many regions outside of China as the NSIDC dataset was used to derive validation values^9,10^. Thereby, validation data for potentially problematic regions was not included which may mask uncertainties and the strong negative effect of poor station data availability on the performance of gridded products. The additional assessment using evaluation stations not included in the *GPCC* dataset during the 1998–2012 period, with simultaneous error increases in most products, further illustrated the positive bias of including non-independent station values and only left three products with better performance than the mean station value according to *Eff* (MERRA-2 BC, GPCC Full Data Product, GPCC Monitoring Product). Surprisingly, also most reanalysis products were substantially affected by excluding Khorog station data from the evaluation. This is in agreement with the spatial assessment, not only indicating a very strong influence of station data availability on GPCC products, with lower errors at the stations located next to the GPCC grid cell with regular station data input and similar precipitation regimes, but also an indirect influence on spatial performance of many other gridded products deriving information from the *GPCC* dataset (*GPCP*, *PERSIANN-CDR*, *TRMM*). For several datasets (*ERA-interim*, *MERRA-2 BC*), the observation data sources are not clear but the error pattern indicates similar integrated station data cf.^55^. Apparently, gauge-based *CRU* shows an analogous pattern with lower errors at locations in proximity to the grid cell with most integrated station data (Khorog). The *CRU* product also led to some unexpected results. No significant performance changes were observed in spite of higher regional station data availability during the 1980–1994 period. The reason for this may be the strong influence of station data from more distant locations with different precipitation regimes. In addition to higher station density, different algorithms of station data integration may also result in strong variations in regional performance of different gauge based datasets hence. This may be one reason for the superior performance of *GPCC* products compared to the *CRU* dataset which was also found in existing research^15,19^. However, it is important to state that much higher station data availability and the comparison of datasets with different densities of integrated gauges may be necessary to evaluate spatial errors in more detail cf.^28^. This underlines that peripheral rural areas are also characterized by limitations in the evaluation of datasets due to poor meteorological infrastructure. Additionally, uncertainties in measuring snowfall and the impact of wind drift produce errors that may considerably influences gauge based evaluation studies in mountainous terrain. Unshielded gauges may result in undercatch errors frequently ranging between 20% and 50% in windy conditions^64^, with extreme values up to 80%^25^. In the research area, Tretyakov precipitation gauges are used for measuring precipitation. Existing studies documented a snow catch-ratio of about 74–77% compared to Double Fence Intercomparison Reference gauges for this type^64,65^. Therefore, different wind patterns and precipitation composition lead to spatially and temporarily variable errors of about 25% in solid precipitation on average. This further increases uncertainties of gauged based products and gauge based evaluations.

Generally, the *GPCC* products were still among the better performing datasets in periods of scarce station data availability which indicates limited ability of the dataset to derive local scale precipitation in periods of low integrated observation values as well. But in contrast to other studies from the region^9,15,18^, the *GPCC Full Data Product* was not the best of the evaluated products and was slightly outperformed by *MERRA-2 BC* during the 1998–2012 period. This shows that a combined product, incorporating reanalysis information and station correction, may be an important alternative to interpolated products in data scarce regions. Nevertheless, the significant performance decrease of MERRA-2 BC in the more recent assessment period may also be an indication that station data availability influences combined products. The station calibrated, satellite based *TRMM* dataset also showed a limited ability to predict local precipitation values which agrees with results obtained by Hu *et al*.^10^. But due to potential effect of similar incorporated station values by utilizing GPCC data, the product may also suffer from overly optimistic gauge based evaluations as shown by the removal of Khorog station from the evaluation set. Although the lack of gauge data for interpolation or calibration resulted in considerable errors with all of the aforementioned products in complex terrain and much higher station density may be needed to capture contrasting precipitation gradients cf.^25,66^, indicating that the datasets are not applicable for many scientific applications, they still showed some capabilities to provide rainfall amounts at the station scale compared to the other evaluated datasets (*CRU*, *GPCP*, *PERSIANN-CDR*, *MERRA-2*, *ERA-interim*, *ERA5*). These products are not applicable without further corrections, data integration or downscaling approaches due to high associated errors. For some datasets such as *GPCP* or *ERA-interim*, spatial resolution may be an explanation for this result but the similar performance of *ERA5* with higher resolution indicates that without further data enhancements, slightly higher spatial resolution may not lead to performance improvements of the reanalysis data. Existing studies indicate that very high resolutions of up to 4–6 km are necessary to accurately model snow and orographic precipitation^67,68^. However, higher correlation values of the reanalysis products in periods of low gauge data availability indicate that they have the potential to predict tendencies of precipitation variations without capturing the magnitude^69^. As reanalysis products simulate average grid cell precipitation and represent a mix of valleys, slopes and peaks and are not affected by undercatch errors, a direct comparison to gauge data is difficult and an appropriate adaption may be necessary. This is roughly illustrated with a simple linear regression model with station data during the 1980–1994 period as the dependent variable and ERA5 data plus the averaged SRTM^70^ elevation in ERA5 pixels at the stations as independent variables. Resulting linear coefficients may be used for adjusting ERA5 data to station values in the later time period. Although independent stations would be preferable for deriving coefficients and errors may be positively biased with this approach, such a model based adjustment would result in considerably lower mean errors for the ERA5 evaluation for 1998–2012 with *MAE* values of 10.25 mm and an *Eff* of 0.37. This theoretical example illustrates that reanalysis data have to be considered as an important alternative compared to gauge based products in data poor regions, although they are characterized by higher precipitation magnitudes. The estimation of averaged precipitation amounts in mountain areas is further complicated by the situation that most climate stations worldwide are positioned in valleys and therefore, do not sufficiently represent high altitude areas.

Similarly, some low resolution products may lead to situations with more than one station located within a pixel. Respective datasets may be slightly better described by station averages in contrast to several single stations within the pixel. To assess potential effects, we additionally averaged stations falling within a pixel in the dataset with the coarsest resolution (GPCP) and performed another evaluation. The resulting Eff of 0.02 for GPCP indicates a positive effect of pixel averaging of stations for low resolution products, although the variability with low station numbers is relatively high.

Temporarily, *ERA-interim*, *ERA5* and *PERSIANN-CDR* also showed significant performance improvements in the 1998–2012 period. Regarding *ERA* products, the reason for this progress is most likely due to a strong increase of integrated observation and aircraft data in recent decades^54^. Lower performance of *PERSIANN-CDR* in the pre-1997 period can be explained by a limited number of passive/active microwave observations aboard low earth orbit satellites^52^. Respective results indicate that improved data availability is also essential for significant improvements of reanalysis and satellite based datasets. Finally, we showed that simple outlier correction algorithms are capable of increasing the performance of gauge data or satellite based products substantially, but may also decrease their performance if enough station data was included originally. On the other hand, outlier correction had no or only limited effects for most products and more complex approaches, such as bias-correction methods^71,72^, may be necessary to improve the various datasets. However, as respective approaches require independent gauge data, they are not applicable without increasing climate station infrastructure in data scarce regions. The homogeneity assessment also indicated that breaks are present in one dataset or in some regions. MERRA-2 BC had a major break during 1993–1996 which may be caused by a reduction of station data available for the incorporated CPC product due to the onset of the civil war. The break in the Murghab time series of GPCP and PERSIANN-CDR may be a result of generally missing station values for the correction algorithm from this station during the respective years. So most inhomogeneities may be caused by a variability in the station network cf.^28^ which may partly explain temporal performance changes.

In conclusion, this study provides evidence that scarce station data has profound effects on the performance of several gridded precipitation products in complex terrain as most datasets are characterized by direct or indirect dependencies on observation networks. Substantial error increases in periods of low data availability illustrate the need for evaluating the spatial and temporal pattern of integrated observation data before respective products are utilized. Otherwise, precipitation values from gridded datasets cannot be reasonably evaluated and may be unsuitable for scientific applications. Temporarily, datasets using station based gauge data observations showed a decline in performance in the Pamir mountains of Central Asia during more recent periods whereas most reanalysis and satellite products with higher resolution improved significantly. Future research may greatly benefit from increased efforts to combine or adapt several gridded precipitation sources to derive surface precipitation amounts in complex terrain similar to existing approaches such as the merged MSWEP 1979–2015 product^73^. DIVA-GIS^74^, QGIS^75^.

## Data Availability

All utilized raster datasets, NEESPI and NSIDC station data are available for download free of charge from the respective sources. Station data from the SAHRT may be obtained from the respective ministry and we are not entitled to provide respective data online.
